# Ultrafast Breast MRI: A Narrative Review

**DOI:** 10.3390/jpm15040142

**Published:** 2025-04-02

**Authors:** Ottavia Battaglia, Filippo Pesapane, Silvia Penco, Giulia Signorelli, Valeria Dominelli, Luca Nicosia, Anna Carla Bozzini, Anna Rotili, Enrico Cassano

**Affiliations:** Breast Imaging Division, Radiology Department, IEO European Institute of Oncology IRCCS, Via Giuseppe Ripamonti 435, 20141 Milan, Italy; filippo.pesapane@ieo.it (F.P.); silvia.penco@ieo.it (S.P.); giulia.signorelli@ieo.it (G.S.); valeria.dominelli@ieo.it (V.D.); luca.nicosia@ieo.it (L.N.); anna.bozzini@ieo.it (A.C.B.); anna.rotili@ieo.it (A.R.); enrico.cassano@ieo.it (E.C.)

**Keywords:** magnetic resonance imaging, breast MRI, ultrafast breast MRI, breast cancer

## Abstract

Breast magnetic resonance imaging (MRI) is considered the most effective method for detecting breast cancer due to its high sensitivity. Yet multiple factors limit its widespread use, including high direct and indirect costs, a prolonged acquisition time with consequent patient discomfort, and a lack of trained radiologists. During the last decade, new strategies have been followed to increase the availability of breast MRI, including the omission of non-essential sequences to generate abbreviated MRI protocols (AB-MRIs) aimed at reducing the acquisition time with the potential of improving the patient’s experience and accommodating a higher number of MRI examinations per day. An alternative method is ultrafast MRI (UF-MRI), a novel technique that gathers kinetic data within the first minute after contrast injection, offering high temporal resolution. This enables the analysis of early contrast wash-in curves, showing promising outcomes. In this study, we reviewed the role of UF-MRI in breast imaging and detailed how the integration of this new approach with radiomics and mathematical models might further improve diagnostic accuracy and even have a prognostic role, a fundamental characteristic in the modern scenarios of personalized medicine. In addition, possible clinical applications and advantages of UF-MRI will be discussed.

## 1. Introduction

Breast magnetic resonance imaging (MRI) is the most sensitive technique to detect breast cancer, with its main indications, according to the European Society of Breast Cancer Specialists (EUSOMA) working group, being staging before treatment planning, evaluation of treatment response to neoadjuvant chemotherapy and screening of high-risk women, among others [1].

Dynamic contrast-enhanced breast MRI (DCE-MRI) optimizes the distinction between the lesion and the surrounding glandular tissues and allows kinetic analysis, improving the specificity of the exam, with a negative predictive value exceeding 90% [2,3]. The traditional kinetic analysis in DCE-MRI, which captures high-spatial-resolution images of the entire breast, involves creating time–intensity curves from pre-contrast, early- and delayed-phase images [4].

A full protocol of dynamic contrast-enhanced breast MRI (FP-DCE MRI) typically lasts between 12 and 20 min: in most facilities, only two breast MRI exams are conducted per hour on a single MRI scanner [5]. Thus, despite the well-established role of the FP-DCE MRI, the relatively long acquisition time, together with the high direct and indirect costs and patient discomfort, remain key issues [6].

In this scenario, the trendingapproach has been to streamline breast MRI protocols by eliminating non-essential sequences in order to generate abbreviated MRI (AB-MRI) examinations. This modification shortens the scan time to approximately three minutes per exam, relying solely on the first post-contrast image [7]. Although several studies [8,9,10,11] and meta-analyses [12,13] have demonstrated the high diagnostic accuracy of AB-MRI, the lack of kinetic information is one main limit.

A possible solution might be represented by ultrafast MRI (UF-MRI), a recent technique which captures kinetic information within the first minute after contrast injection using a high temporal resolution (3–10 s).

UF-MRI, instead of relying on the conventional washout characteristics, allows the analysis of early contrast wash-in curves, reflecting the contrast inflow effects.

The combination of an abbreviated and an ultrafast protocol has not yet been extensively studied, but it represents a potentially promising approach to meeting the need for a dynamic scan protocol without significantly increasing imaging time [14]. In this narrative review, we aim to explore the current state of the art of the ultrafast protocol in breast MRI.

## 2. Ultrafast Imaging Technique

For UF-MRI, either 1.5 T or 3 T scanners equipped with dedicated breast coils can be used.

Ultrafast sequences, such as time-resolved angiography with stochastic trajectories (TWIST) [15] or 3D dual-echo fat–water-separated T1-weighted differential subsampling with Cartesian ordering (DISCO) [16] sequences, can be used in oder to monitor the early contrast inflow of breast lesions.

The TWIST technique enables rapid 3D dynamic imaging by utilizing a view-sharing approach, alternating between capturing two k-space regions: the central region (A) and the peripheral region (B) [15].

The DISCO sequence features an elliptically ordered k_y_-k_z_ space that is divided into N annular regions. Each region, “i”, undergoes subsampling at a factor of Si (typically, Si ≥ i), where the central region is fully sampled, while the outer regions are progressively subsampled by retaining every S_i_^th^ sample [16].

These ultrafast sequences have a high temporal resolution, sometimes at the expense of a lower spatial resolution, as these techniques heavily undersample the outer part of k-space; nevertheless, data points can be shared between consecutive time points, enhancing spatial resolution to a level suitable for diagnostic quality.

Ultrafast MRI can also be performed using a prototype TWIST-VIBE sequence based on the 3D gradient-echo volumetric interpolated breath-hold examination (VIBE) sequence, combined with Dixon water-fat separation (TVD) or with compressed-sensing (CS) reconstruction (CS-VIBE). The CS-VIBE approach can achieve both high spatial and temporal resolution with imaging parameters of a repetition time/echo time of 5.0/2.5 ms, a flip angle of 15°, a field of view of 360 × 360 mm, a 384 × 269 matrix, a slice thickness of 2.5 mm, a CS acceleration factor of 16.5, a temporal resolution of 3.7 s per frame and 20 frames per acquisition [17,18,19].

Ohashi et al. [20] introduced an ultrafast DCE-MRI protocol utilizing k-space-weighted image contrast (KWIC) acquired within the first minute after contrast injection. The KWIC sequence employs a radial k-space acquisition combined with view sharing, enabling high-temporal-resolution imaging [20].

There is no consensus yet on the optimal duration of a UF-MRI scan, and its standardization remains an open question. A study by Cao et al. [21] aimed to identify the ideal scan duration for distinguishing between benign and malignant breast lesions. Their findings suggest that a scan duration of 67.5 s provides the best balance between imaging efficiency and diagnostic accuracy [21].

All the technical parameters of the different ultrafast sequences are specified in Table 1.

## 3. Exam Interpretation

Among the various parameters that can be derived from a UF-MRI examination, maximum slope (MS) and time to enhancement (TTE) are among the most frequently analyzed.

MS, introduced by Mann et al. [22] in 2014, quantifies the time–intensity curve by identifying the steepest part of the enhancing curve within the first minute. It is calculated as the percentage change in relative enhancement per second (%/s). Their study demonstrated that MS has a significantly higher ability to distinguish between benign and malignant lesions compared to the breast imaging reporting and data system (BI-RADS) curve types, with areas under the curve (AUCs) of 0.829 and 0.692, respectively, using histopathological reports or lesion stability for more than two years as reference points [22].

TTE, introduced by Mus et al. [23], determines the earliest moment of lesion enhancement. It is calculated by subtracting the “time at which the aorta starts enhancing” from “the time at which the lesion first enhances” on maximum-intensity projection (MIP) images. In their study, Mus et al. [23] considered the onset of aortic enhancement as time zero and defined the first MIP image in which the lesion became visible as the lesion’s enhancement onset. By analyzing a total of 195 enhancing lesions, they showed that TTE exhibited significantly better discriminative ability than curve type, with *p*-values of <0.001 and 0.026 for readers 1 and 2, respectively. Additionally, specificity and diagnostic accuracy were notably higher for TWIST compared to VIBE assessment (*p* < 0.001). Moreover, inter-reader agreement in distinguishing malignant from benign lesions was nearly perfect for TWIST evaluation (κ = 0.86) and substantial for conventional assessment (κ = 0.75) [23].

Both MS and TTE reflect the same pathophysiological mechanism (AV shunting and capillary leakage), and, for this reason, several studies have reported no statistically significant difference in terms of diagnostic accuracy between these two methods, as shown in the meta-analysis performed by Amitai et al. [24].

Additional UF-MRI-derived parameters include the bolus arrival time (BAT), which represents the duration from the start of contrast injection to the tracer bolus arrival at a lesion. Another parameter is the initial area under the gadolinium contrast-agent concentration–time curve (IAUGC), calculated as the ratio of the area under the tissue concentration curve from BAT to 60 s post-injection to the area under the arterial input function concentration curve over the same time frame. These parameters, together with conventional DCE-MRI-derived parameters, were analyzed in a study conducted by Onishi et al. [25], who demonstrated that biopsy-proven carcinomas had a significantly larger contrast-enhanced ratio (CER), MS and IAUGC and shorter BAT than benign lesions.

Furthermore, the high temporal resolution achieved with UF-MRI using compressed sensing allows for the distinct visualization of breast arteries and veins, enabling the analysis of the time interval between arterial and venous visualization (A-V interval), which is not possible with conventional DCE-MRI. Onshi et al. [25] reported that A-V intervals for vessels on the tumor-affected side were significantly shorter than those on the non-affected side. This may be attributed to the tumor’s heterogeneous vascular network, which can develop shunts—short, low-resistance, high-flow pathways [26,27,28].

Abe et al. [29] used the initial enhancement rate and the signal enhancement ratio (SER) as parameters to compare diagnostic utility for differentiating benign from malignant lesions between UF-MRI and conventional DCE-MRI. They identified statistically significant differences in signal enhancement ratios (SERs) (*p* = 0.0001) and initial enhancement rates (*p* = 0.0014) between malignant and benign lesions. These findings suggest that ultrafast imaging parameters are valuable in distinguishing between benign and malignant lesions, offering a diagnostic utility comparable to standard kinetic assessment but within a shorter acquisition time [29].

Table 2 synthesizes the different parameters analyzed with UF-MRI.

## 4. Radiomics and Mathematical Model Application

The complex nature of the information obtained from the different breast MRI sequences translates into a difficult and labor-intensive interpretation process. Moreover, the inter-observer variability remains a key issue in the diagnostic process, and it is strongly influenced by the level of the reader’s expertise.

The implementation of computer-aided diagnosis (CAD) systems can enhance diagnostic accuracy by minimizing intra- and inter-observer variability while also assisting in clinical decision-making [30,31].

The study published by Platel et al. [32] was among the first studies that explored the use of a CAD system in the setting of UF-MRI. Their purpose was to investigate how features extracted from UF-MRI analysis measure up to the features used for conventional DCE-MRI in the distinction between benign and malignant lesions.

The performances of the features extracted from both UF-MRI and conventional DCE-MRI were measured by a CAD, and lesion classification was performed with four different classifiers: a k-nearest neighbor (*k*NN) classifier, a linear support vector machine (SVM) classifier, an SVM classifier with a radial basis function (RBF) kernel, and a random forest (RF) classifier. For all four classifiers, the classification performance of kinetics derived from UF-MRI was shown to be significantly superior to that of kinetics obtained from conventional DCE-MRI. This suggests that the high temporal resolution of UF-MRI provides a more detailed depiction of contrast-agent uptake, offering greater diagnostic information than conventional kinetic curves, despite the lack of washout characterization in ultrafast acquisitions [32].

With the advances made in the field of medical image analysis during the last decade, alwaysmore pattern recognition tools and larger in-data set size have been seen. These, in turn, have enabled the development of methods for high-throughput extraction of quantitative features, transforming medical images into analyzable data. The subsequent analysis of these data for decision support has given rise to the emerging field of radiomics [33].

Radiomics, employing data of first-, second- and higher-order statistics, by combining image-derived data with other patient data, it is capable of developing models that may have diagnostic, prognostic and predictive roles [34].

Up to now, very few studies have investigated the diagnostic efficiency of radiomic features on ultrafast imaging.

Lyu et al. [35] conducted a study to evaluate whether integrating radiomic features extracted from ultrafast MRI with an artificial neural network (ANN) could enhance the differentiation of BI-RADS 4 breast lesions, a category with a malignancy probability between 2% and 95% [36]. The objective was to reduce false positives and avoid unnecessary biopsies. In the study, an experienced radiologist utilized freely available software (FAE) to extract radiomic features, which were then refined through principal component analysis (PCA). A multilayer perceptron (MLP) ANN was subsequently used to develop predictive models, with PCA-selected components as inputs and histological findings as the gold standard for malignancy assessment. The ANN models demonstrated outstanding diagnostic performance, achieving AUC values between 0.915 and 0.956. High-sensitivity cutoffs derived from the training dataset suggested that unnecessary biopsies could be reduced by 63.33% to 83.33%, with only one false-negative case in the testing dataset [35].

One of the key advantages of UF-MRI is its ability to track contrast-media bolus propagation through arteries and veins, allowing for a detailed quantitative analysis of breast vasculature morphology and function. This capability was highlighted in a study by Wu et al. [37], where a segmentation technique was used to differentiate vasculature from surrounding tumor tissue. This approach, based on a fuzzy c-means (FCM) method developed by Giger et al. [38] and enhanced with a Hessian filter, demonstrated the potential of UF-MRI in providing accurate breast cancer diagnoses through vascular characterization. These findings suggest that integrating UF-MRI with advanced computational models could further refine breast cancer detection and reduce unnecessary interventions.

By means of their method, the authors created a probability map for each voxel indicating its likelihood of belonging to a vessel. The segmented vessels were then skeletonized and transformed into a graph, ultimately leading to a 3D reconstruction of the breast vasculature. This approach enabled the identification of “tumor-associated vessels”. They then combined morphological information with pharmacokinetic data. For the pharmacokinetic analysis, they chose a simplified version of the standard Tofts–Kety model. The statistical analysis showed that the vessel count, the volume transfer coefficient (K_trans_) and the plasma volume fraction (v_p_) showed significant differences between malignant and benign lesions and that K_trans_ and v_p_ were significantly correlated [37]

Peter et al. [17] investigated the integration of diffusion-weighted imaging (DWI) features with a generalized linear model (GLM) based on ultrafast imaging parameters to enhance diagnostic accuracy in breast lesion classification. Their study demonstrated that the total variation denoising (TVD) model significantly outperformed peak enhancement as a standalone predictor (AUC: 0.938 vs. 0.781, *p* = 0.008) and was also superior to the dynamic contrast-enhanced (DCE) curve type as a singular predictor (AUC: 0.700, *p* = 0.002). These findings suggest that combining ultrafast dynamic sequences with DWI in a GLM framework enables highly accurate breast lesion classification while maintaining a sensitivity and specificity comparable to conventional DCE imaging. This approach may offer a more refined diagnostic tool, potentially improving the accuracy of breast cancer assessments and supporting more precise treatment planning [17].

Mori et al. [39] conducted a study to determine whether parameters derived from a truncated empiric mathematical model (EMM) for enhancement in the very early post-contrast phases of UF-MRI correlated with histological microvessel density (MVD) or vascularity in invasive breast cancer. They quantitatively analyzed the kinetic curve from ultrafast images using the truncated EMM and found that several parameters—the upper limit of signal intensity (A), the rate of signal increase (α), the initial slope of enhancement (Aα), AUC30 and the time of initial enhancement (TIE)—exhibited near-perfect inter-observer reliability and significant correlations with histopathological MVD [39]. Since MVD is associated with metastasis risk, prognosis and response to neoadjuvant chemotherapy, assessing it through UF-MRI with the truncated EMM could be valuable for predicting prognosis and optimizing treatment strategies [40,41,42,43,44,45,46,47].

It is evident that ultrafast imaging within the first minute of breast DCE-MRI can provide valuable insights into early contrast dynamics. Pineda et al. [48] conducted a study to analyze the kinetics of early enhancement in arteries, veins, and malignant and benign lesions, as well as normal-appearing parenchyma. Their study also assessed the effectiveness of early kinetic parameters in distinguishing malignant from benign lesions. Specifically, the percent signal enhancement over time was modeled using a truncated (uptake-only) empiric mathematical model (EMM) for malignant lesions, benign lesions and parenchyma. Based on the EMM parameters, three secondary parameters were derived: the initial area under the contrast enhancement-versus-time curve (iAUC), the time to reach 90% of maximum enhancement (T90) and the initial slope (calculated as the product of the uptake rate and the upper limit of enhancement). The EMM was also used to refine the estimate of lesion time of arrival (TOA), and the authors reported that the average TOA for malignant lesions was significantly shorter than the TOA for benign lesions [48].

These studies have shown that ultrafast imaging enables the assessment of local vascular characteristics while minimizing the impact of global variables, such as cardiac output, and this, in turn, has the potential to allow radiologists to confidently identify lesions, even in suboptimal diagnostic conditions, as the presence of marked background parenchymal enhancement.

## 5. UF-DCE MRI and Possible Clinical Applications

The analysis of kinetic parameters derived from UF-DCE MRI enables precise differentiation of breast lesions, making it highly clinically significant. In addition to the diagnostic setting, UF-MRI has been studied for the follow-up of women with a personal history of breast cancer and in the setting of neoadjuvant therapy response evaluation.

Kim et al. [49] investigated the added value of ultrafast MRI in abbreviated breast MRI (AB-MRI) for the surveillance of women with a personal history of breast cancer. Their findings indicated that incorporating UF-DCE MRI significantly improved specificity (95.3% vs. 88.6%, *p* < 0.001, for all readers) and positive predictive value 1 (PPV1) (21% vs. 10%, *p* < 0.001, for all readers) compared to AB-MRI alone. This enhancement reduced unnecessary short-term follow-ups while maintaining sensitivity in post-operative surveillance [49].

In the context of neoadjuvant chemotherapy (NAC), primarily aimed at reducing tumor size to enable less invasive surgical options, the absence of residual invasive cancer or affected lymph nodes following this therapy is referred to as a pathologic complete response (pCR). The response to NAC is a key prognostic factor linked to improved long-term disease-free survival and overall survival rates [50].

Accurately predicting a patient’s response to NAC before treatment can aid in selecting the most appropriate therapy, reducing overall healthcare costs and minimizing unnecessary exposure to chemotherapy-related toxicity [51].

Ren et al. [52] explored whether assessing the bilateral asymmetry of semiquantitative (MaxSlope, AUC30, TTMS and BAT) and quantitative (Ktrans and Ve) perfusion parameters from UF-MRI could facilitate early prediction of pCR in patients with HER2+ breast cancer. Their findings indicated that bilateral asymmetry in background parenchymal enhancement kinetic parameters (kBPEs), specifically the transfer constant (Ktrans), the ipsilateral/contralateral (I/C) ratio and the extravascular extracellular space fractional volume (Ve) I/C ratio of the top 10% enhancing parenchymal voxels from pre-NAC UF-MRI, significantly differentiated patients who achieved pCR and those who did not (*p* < 0.05). Those who attained pCR after NAC exhibited lower I/C ratios in MaxSlope, AUC30 and Ktrans, as well as smaller differences in time to maximum slope (TTMS) and BAT between the two breasts in pre-NAC scans. Moreover, a pronounced disparity in BPE kinetics between the affected and contralateral breast, observed in pre-NAC MRI scans, was strongly correlated with residual disease post-NAC. Therefore, both semiquantitative and quantitative pharmacokinetic parameters from pre-NAC UF-MRI may serve as independent predictive markers of pCR in patients with HER2+ breast cancer [52].

On the other hand, Honda et al. [53] investigated the diagnostic performance of UF-MRI following the completion of neoadjuvant chemotherapy (NAC) in breast cancer. The results showed that UF-MRI achieved a higher AUC and specificity compared to full-protocol DCE-MRI, enabling more accurate detection of residual cancer and better visualization of tumor extent. Residual tumor sizes were assessed using the 20th phase of UF-MRI, as well as the early and delayed phases of conventional DCE-MRI and high-spatial-resolution MRI. UF-MRI demonstrated the highest overall AUC, with values of 0.86 and 0.88 for readers 1 and 2, respectively [53].

A study conducted by Choi et al. [54] explored the potential role of kinetic parameters derived from UF-MRI in predicting pCR after NAC and examined their correlation with histologic microvessel density (MVD). They highlighted that MRI kinetic parameters were generally less influential in predicting pCR across all breast cancer subtypes compared to the clinical stage of the tumor and molecular characteristics. Nevertheless, in HER2-enriched breast cancer, a significant difference in early kinetic parameters was observed between the pCR and non-pCR groups, independent of clinical stage. Specifically, in the HER2-enriched subgroup, patients who achieved pCR had a lower initial enhancement value (median: 349.0% [224.0–778.0] vs. 664.0% [344.0–1332.0], *p* = 0.04) and a smaller CAD angiovolume (3.2 cc [2.2–9.6] vs. 10.9 cc [4.1–104.0], *p* = 0.04) compared to those who did not achieve pCR. These findings suggest that the predictive significance of MRI kinetic parameters varies by molecular subtype, potentially paving the way for new advancements in personalized medicine.

Further research is necessary to validate and establish specific MRI kinetic parameters associated with pCR in different breast cancer subtypes. This would help refine treatment decisions, enhance patient outcomes and reduce unnecessary treatment exposure. As precision oncology continues to evolve, integrating UF-MRI into clinical practice could play a crucial role in optimizing patient management and ensuring that individuals receive the most appropriate and effective therapy.

## 6. Ultrafast Breast MRI in the Era of Personalized Medicine

The paradigm of personalized medicine aims to tailor medical decisions and treatments to the individual characteristics of each patient, integrating clinical, radiological, histopathological, and, increasingly, molecular and imaging-derived data [55]. In breast imaging, personalized approaches are becoming increasingly relevant to stratify patient risk, select optimal screening modalities, predict therapeutic response and monitor disease evolution [56].

The wealth of quantitative information derived from UF-MRI has correlations with histopathological features, such as microvessel density, tumor subtype and response to neoadjuvant chemotherapy, supporting their potential prognostic and predictive value [57].

Moreover, the integration of UF-MRI with advanced mathematical modeling, AI and radiomics facilitates the high-throughput extraction of features that go beyond human perception [58]. These features can be incorporated into machine learning algorithms or clinical decision support systems to predict malignancy, treatment response or recurrence risk [59]. For example, combining radiomics extracted from UF-MRI with artificial neural networks has been shown to significantly reduce false-positive biopsies in BI-RADS 4 lesions while maintaining high diagnostic accuracy [35].

Additionally, UF-MRI-derived kinetic parameters can quantify inter-breast asymmetries in background parenchymal enhancement [60], which may serve as early imaging biomarkers to predict responses in patients undergoing neoadjuvant chemotherapy [61]: this capability supports more precise therapeutic stratification and potentially facilitates treatment de-escalation in responders [62].

In the evolving landscape of breast cancer care, where early detection, risk-adapted screening and individualized treatment planning are prioritized, UF-MRI stands as a valuable imaging tool that aligns with the principles of precision medicine. Its incorporation into multiparametric and multimodal frameworks may ultimately contribute to a more nuanced and individualized approach to breast cancer diagnosis and management [6,7].

## 7. Conclusions

Contrast-enhanced breast MRI is the most sensitive technique to investigate breast cancer, with reported sensitivities being even more than 95% in expert centers worldwide [5,63].

On the other hand, a major drawback is the high rate of false-positive results, leading to a significant number of unnecessary biopsies. This not only imposes psychological stress on patients but also contributes to increased financial burdens [64].

Thus, the combination of morphological and functional information related to lesion vasculature could enhance diagnostic accuracy beyond what can be achieved by either approach individually.

This analysis can be performed with the support of mathematical models and radiomics in order to further enhance diagnostic accuracy and speed up the evaluation process. Moreover, the promising results of UF-MRI-derived parameters as prognostic factors in patients receiving neoadjuvant chemotherapy might open new prospects in the setting of personalized medicine, serving as a base for tailored patient treatment and ad hoc follow-up strategies.

## Figures and Tables

**Table 1:** Technical parameters of UF-DCE MRI.

Ultrafast Imaging Technique/Parameter	TWIST-VIBE	CS-VIBE	DISCO	TVD	KWIC
Acquisition time (s)	78	75	60	98	60
TR/TE (ms)	6.89/2.4	5/2.5	3.8/1.7	5.64/2.46	3.57/1.68
Voxel size (mm)	1.38 × 1.17 × 2	N/A	N/A	0.9 × 0.9 × 2.5	N/A
FoV (mm)	375 × 300	360 × 360	212 × 212	360 × 360	333 × 330
Flip angle (°)	15	15	10	15	15
Slice thickness (mm)	2	2.5	1.6	2.5	2.5
Fat suppression method	TWIST-VIBE with Dixon fat suppression	VIBE with fat suppression	N/A	Dixon fat–water separation	VIBE with fat suppression
No. of slices	72	60	N/A	60	60

TWIST (time-resolved angiography with stochastic trajectories); VIBE (volumetric interpolated breath-hold examination); CS-VIBE (compressed-sensing volumetric interpolated breath-hold examination); DISCO (dual-echo fat–water-separated T1-weighted differential subsampling with Cartesian ordering); TVD (TWIST-VIBE sequence with Dixon water–fat separation); KWIC (k-space-weighted image contrast); TR/TE (repetition time/echo time); FoV (field of view).

**Table 2:** Parameters of UF-MRI.

Parameter	Details	Unit of Measurement
MS (Maximum Slope)	The relative enhancement percentage change of the tangent along the steepest part of the enhancing curve within the first minute divided by seconds.	%/s
TTE (Time to Enhancement)	The time point at which the lesion starts to enhance minus the time point where the aorta starts to enhance.	s
BAT (Bolus Arrival Time)	The time from the start of contrast injection to tracer bolus arrival at a lesion.	s
IAUGC (Initial Area Under the Gadolinium Contrast-Agent Concentration–Time curve)	The area under the tissue concentration curve from BAT to 60 s from the start of contrast injection divided by the area under the arterial input function concentration curve from BAT to 60 s from the start of contrast injection.	mmol/s
A-V interval (Time Interval between Arterial and Venous Visualization)	Time interval between arterial and venous visualization.	s
SER (Signal Enhancement Ratio)	(S1 − S0)/(S2 − S0), where S0 is the signal intensity pre-contrast, S1 is the signal intensity at early post-contrast and S2 is the signal intensity at late post-contrast.	s

## Data Availability

Not applicable.
